# Exploring determinants of self-management in adults with severe mental illness: a qualitative evidence synthesis

**DOI:** 10.1192/bjo.2021.93

**Published:** 2021-06-18

**Authors:** Claire Carswell, Jennifer Brown, Abisola Balogun, Jo Taylor, Peter Coventry, Charlotte Kitchen, Ian Kellar, Emily Peckham, Sue Bellass, Sarah Alderson, Jennie Lister, Richard Holt, Catherine Hewitt, Rowena Jacobs, David Shiers, Jan Boehnke, Ramzi Ajjan, Najma Siddiqi, On behalf of the DIAMONDS Research team

**Affiliations:** 1 University of York; 2 University of York, Hull York Medical School; 3 University of Leeds, School of Psychology; 4 University of York, University of Leeds, School of Medicine; 5 University of Leeds, School of Medicine; 6 University of Southampton, University Hospital Southampton NHS Foundation Trust; 7 University of Manchester, Psychosis Research Unit, Greater Manchester Mental Health NHS Foundation Trust; 8 School of Health Sciences, University of Dundee

## Abstract

**Aims:**

To systematically review and synthesise qualitative evidence about determinants of self-management in adults with SMI. The goal is to use findings from this review to inform the design of effective self-management strategies for people with SMI and LTCs.

**Background:**

People living with serious mental illness (SMI) have a reduced life expectancy by around 15–20 years, mainly due to the high prevalence of long-term physical conditions such as diabetes and heart disease. People with SMI face many challenges when trying to manage their physical health. Little is known about the determinants of self-management – managing the emotional and practical issues – of long-term conditions (LTCs) for people with SMI.

**Method:**

Six databases, including CINAHL and MEDLINE, were searched to identify qualitative studies that explored people's perceptions about determinants of self-management in adults with SMI (with or without comorbid LTCs). Self-management was defined according to the American Association of Diabetes Educator's self-care behaviours (AADE7). Determinants were defined according to the Capabilities, Opportunity, Motivations and Behaviours (COM-B) framework. Eligible studies were purposively sampled for synthesis according to the richness of the data (assessed using Ames et al (2017)'s data richness scale), and thematically synthesised.

**Result:**

Twenty-six articles were included in the synthesis. Seven studies focused on self-management of LTCs, with the remaining articles exploring self-management of SMI. Six analytic themes and 28 sub-themes were identified from the synthesis. The themes included: the additional burden of SMI; living with comorbidities; beliefs and attitudes about self-management; support from others for self-management; social and environmental factors; routine, structure and planning. Capabilities for self-management were linked to people's perceptions about the support they received for their SMI and LTC from healthcare professionals, family and friends. Opportunities for self-management were more commonly expressed in the context of social and environmental factors. Motivation for self-management was influenced by beliefs and attitudes, whilst being closely related to the burden of SMI.

**Conclusion:**

The themes identified from the synthesis suggest that capabilities, opportunities and motivations for self-management can be negatively influenced by the experience of SMI, whilst social and professional support, improved access to resources, and increased involvement in care, could promote self-management. Support programmes for people with SMI and LTCs need to account for these experiences and adapt to meet the unique needs of this population.

